# Cultural adaptation, translation, and validation of the Measure of Indigenous Racism Experiences Scale for Indigenous gay and bisexual men in Guatemala

**DOI:** 10.3389/fpubh.2026.1864458

**Published:** 2026-07-13

**Authors:** Catherine Purnell, José Yac, Brandon Merida, Joao Ricardo Nickenig Vissoci, E. Roberto Orellana, Dirk A. Davis

**Affiliations:** 1 Wake Forest School of Medicine, Wake Forest University, Winston-Salem, NC, United States; 2 Duke Global Health Institute, Duke University, Durham, NC, United States; 3 Asociación IDEI, Quetzaltenango, Guatemala; 4 Trabajando Unidos NGO Huehue, Huehuetenango, Guatemala; 5 School of Social Work, University of Washington, Seattle, WA, United States

**Keywords:** gay and bisexual men, Guatemala, Indigenous people, item variance, systemic racism

## Abstract

**Introduction:**

Among Indigenous communities, racism is associated with negative health effects, including worsened physical and mental health outcomes and less engagement with health services. Despite nearly half of Guatemalans identifying as Indigenous, there have been no scales developed and validated to measure self-reported racism in Indigenous populations in Guatemala, nor elsewhere in Latin America. This study validates the Measure of Indigenous Racism Experiences (MIRE) scale, originally developed to measure racism experiences with Aboriginal populations in Australia, for use in Guatemala among Indigenous gay and bisexual men (GBM) population. The MIRE scale uses 16 items to measure three dimensions of self-reported racism: internalized, systemic, and interpersonal.

**Methods:**

The MIRE scale was translated into Spanish and tested with 395 Indigenous GBM in Guatemala. Construct validity was tested through confirmatory factor analysis using the three predetermined dimensions of racism. Three previously reported associations between dimensions were used to test the external validity and were confirmed using cross-tabulations and chi-square tests. Reliability of the scale was tested using Cronbach's alpha. A modification index was used to determine any necessary changes to item categorization within the dimensions.

**Results:**

After removing one scale item and reorganizing another in line with a modification index, the MIRE-Guatemala scale showed good internal validity (CFI = 0.901, SRMR = 0.066, RMSEA = 0.078, 90%CI: 0.067, 0.089). One of the hypothesized trends in the new MIRE-Guatemala scale was confirmed, and another was supported, indicating mixed results for external validity. The Cronbach's alpha for the three dimensions of racism all showed good reliability (α = 0.75, 0.79, 0.86).

**Discussion:**

The MIRE-Guatemala scale showed good internal validity and reliability to be validated for use among Indigenous GBM in Guatemala. External validity is moderate and should be tested further. The validated scale has strong potential to capture the racism experiences of Indigenous GBM in Guatemalan with further research and validation. Research should continue to develop and validate measures to assess Indigenous racism throughout the region.

## 1 Introduction

Research suggests that worldwide, roughly one third of all Indigenous adults experience racism attributed to their Indigenous identity (1). Racism, defined by Paradies, is the “unfair and avoidable disparities in power, resources, capacities, or opportunities centered on ethnic, racial, religious, or cultural differences,” occurring at three levels: internalized, interpersonal, and systemic (1).

Self-reported racism, also known as perceived racism, has negative impacts on both physical and mental health. For example, racism experienced by Indigenous children in the United States, Canada, Australia, and New Zealand is associated with negative outcomes in educational settings, increased substance abuse and conduct disorder, which is characterized by aggressive behavior (2). Racism has been shown to affect mental health, physical health, emotional health, increase maladaptive behaviors, decrease healthy behaviors, and increase exposure to risk factors for disease, among other impacts (1, 3).

Latin America has one of the highest proportions of Indigenous populations in the world with an estimated 45 million individuals identifying as Indigenous (4). In Latin America, racism based on Indigenous identity has been shown to affect income inequality, educational attainment, and other social factors (5). Guatemala, the most populous country in Central America, has a sizeable Indigenous population with 44% of the population identifying as one of 21 Indigenous groups, predominantly Maya (6). Guatemala also has one of the largest disparities in educational attainment between Mestizos (non-Indigenous people) and Indigenous people in all of Latin America and the Caribbean (5). Indigenous Guatemalans have lower educational attainment, are more likely to live in rural areas with fewer medical facilities, are more likely to live in extreme poverty, and encounter language barriers when trying to access medical care and other institutions (7).

Indigenous Guatemalans who also hold other marginalized identities, such as Indigenous gay and bisexual men (GBM), often experience intersectional stigma. Stigma and racism are related, with stigma encompassing the negative stereotyping and power asymmetry that groups face (8), and racism relating to the unequal treatment and/or oppression of individuals based on unfounded beliefs of racial inferiority (9). Both stigma and racism can be based on ethnic or racial identities and have harmful impacts on mental and physical health (8, 9).

In qualitative interviews with Indigenous GBM in Guatemala, our team found that participants frequently experienced stigma from Guatemalan society, including assumptions that Indigenous people were of lower social class and less educated than their Mestizo counterparts (10). Indigenous Guatemalans also reported experiencing racism and stigma more frequently in urban settings, where Mestizos were the majority of the population, taking measures to hide their Indigenous identity in order to experience less violence, and beliefs that the lack of Indigenous representation in government and other institutions was directly tied to the Indigenous identity stigma in the country (10).

Despite overwhelming evidence that racism impacts the health of Indigenous communities in Guatemala and across Latin America, to our knowledge there have been no instruments that measure self-reported racism in Indigenous communities anywhere in Latin America.

Of the few instruments which have been used to measure self-reported racism within Indigenous populations elsewhere, many were one- or two-item measures, including the use of the Negative Life Experiences Scale, which uses perceived racism as one item in the scale (11–13). Other scales were not created specifically for the Indigenous experience (14–19) or were not validated for use (20).

The Measure of Indigenous Racism Experiences (MIRE) scale was created to measure self-reported racism within Australian Aboriginal and/or Torres Strait Islander population and was the first instrument to be validated for use to measure self-reported racism and reactions to racism within Indigenous populations (21). We chose to translate and validate this scale for use in Guatemala among Indigenous GBM populations, as it was created with the specific Indigenous experience in mind and encapsulated all three levels of racism. Other studies have also chosen to use this scale outside Australian Aboriginal populations, including Canadian Aboriginal populations (22) and First Nations of Ontario, Canada (23), though neither provided further validation of the scale for use among these populations.

The aims of this study were to (1) adapt and translate the MIRE scale for use in Spanish with Indigenous GBM in Guatemala, and (2) to perform analysis to ensure reliability, internal structure and external validity of the adapted scale.

## 2 Materials and methods

### 2.1 Participants

The sample is composed of 395 Indigenous Guatemalan GBM as a part of a study which investigated different forms of stigma, mental health, and HIV prevention outcomes among this population in the Western highlands of Guatemala. Participants were included in the study if they were over the age of 18, spoke Spanish, identified as Indigenous, and identified as gay, bisexual or a man who has sex with men. Although there are over 20 Indigenous languages spoken in Guatemala, the majority of young men in Guatemala (our target population) speak Spanish. We, therefore, chose to conduct the survey in Spanish for operational ease.

### 2.2 Instrument

The original MIRE scale is a 31-question scale created to assess self-reported racism in Aboriginal Australians and/or Torres Straits Islanders (21). The 31-items covered three domains of racism: internalized racism, systemic racism and interpersonal racism, as well as reactions and responses to racism, race-consciousness, and salience of Indigeneity within social group and among strangers. The MIRE scale measures the three domains of racism using the average answer to questions, which are asked as a Likert-type scale with responses ranging from 1 to 3, with higher scores indicating more experiences with Indigenous identity racism.

Interpersonal racism was measured using nine items, which asked participants to rate the frequency of mistreatment due to Indigenous identity in various situations. Internalized racism was measured by four items, which assessed participants agreement or disagreement to statements about Indigenous people. Systemic racism was assessed using three items, which measured agreement to statements about treatment of Indigenous people by government and institutions, by other non-Indigenous Guatemalans, and by the media representation. While the MIRE also includes items to measure reactions to racism, we chose to only validate experiences of racism due to concerns over questionnaire length from community partners.

### 2.3 Ethical statement

This study was approved by the ethics committees at Duke University and Universidad de la Valle Guatemala. All participants provided informed consent and were compensated Q125 (USD$12) for their time.

### 2.4 Translation and adaptation

Our Guatemala- and US-based team, including researchers, clinicians, and community outreach specialists, oversaw the translation and adaptation process. The original MIRE scale was translated to Spanish and back-translated to English by four members of the team. Inconsistencies were discussed with the larger team and adjustments were made to finalize content validation. We then piloted the adapted and translated scale with a convenience sample of 23 Indigenous GBM who were participating in qualitative interviews as part of a formative aim for the parent study. After piloting the adapted measure, participants were asked to assess the quality of the questions and coherence of language and content. Minor adjustments were made based on this feedback which led to the final version of the measure used for this study.

### 2.5 Data collection

Data was collected by trained community outreach specialists, the majority of whom identified as Indigenous and gay or bisexual men, at two community organizations, *Asociación para la integración, educación y desarrollo integral* (IDEI), located in Quetzaltenango, Guatemala, and *Trabajando Unidos NGO Huehue*, in Huehuetenango, Guatemala. Both organizations are Indigenous-led and have decades of experience providing preventative health services to Indigenous GBM in Western Guatemala. We used snowball sampling and IDEI and Trabajando Unidos's extensive outreach networks to identify and recruit participants into the study. We used tablets equipped with REDCap Mobile to conduct the cross-sectional survey, which, in addition to the adapted MIRE scale, included scales for other forms of stigma, mental health, and HIV-related outcomes. All surveys were conducted in private locations and lasted between 45 and 60 min.

### 2.6 Data analysis

Sociodemographic data, including age, Indigenous group, department/state of residence, and education level, were collected and reported as mean or count and prevalence. All analyses were done using R software version 4.5.1 licensed under the GNU General Public License. Publically available from: cran.r-project.org/bin/windows/base/old/4.5.1/.

### 2.7 Evidence of validity

Construct validity is a measure of the extent to which the actual scale follows the theoretical hypothesis of the scale (24). Since this was a translation of a previously theoretically and statistically validated scale, a Confirmatory Factor Analysis (CFA) was used to test the underlying structure of the scale. Prior to running the CFA, Q10 and Q13 were reverse coded.

In accordance with the recommendations from Alavi et al. (25), we used four measures of fit to test the scale structure. The chi-square test is often too sensitive to accurately measure larger sample sizes, and therefore the chi-square test used a *x*^2^/df ratio as the cut-off value rather than a *p*-value of significance (25). A *x*^2^/df ratio of ≤ 5 is considered adequate for a large sample size (26). We also used a Root Mean Square Error of Approximation (RMSEA), with a cut-off of ≤ 0.08, considered a fair fit by MacCallum et al. (27). Further, a Comparative Fit Index (CFI), with a cut-off at ≥0.90, indicating an “acceptable fit,” and a Standardized Root Mean Square Residual (SRMR) with a cut-off ≤ 0.08 were used (28).

A modification index was used to determine which variables needed to be rearranged or removed, within theoretical acceptance, to improve model fit, until the cut-offs are reached (29).

External validity (criterion validity) was tested by using theoretical trends in the data that were indicated in the creation of the original MIRE scale. Paradies and Cunningham (21) hypothesized that internalized and systemic racism would be inversely correlated, higher levels of interpersonal racism would be associated with lower levels of systemic racism, and higher levels of interpersonal racism would be associated with lower levels of internalized racism. Each of the three hypotheses were tested using cross tabulations and chi-square tests, using a *p*-value threshold of 0.05. Additionally, each of the hypothesized trends had to show a consistent linear trend, no cells with 0 values, and less than 20% of all cells with values less than 5 (21).

### 2.8 Evidence of reliability

Internal reliability tests the consistency of the measure. Each dimension of the scale was tested using Cronbach's alpha, with the range for good internal reliability between 0.70 and 0.90 (30).

## 3 Results

### 3.1 Sample characteristics

Of the 395 participants, most had completed high school (50%), over a quarter (28%) completed university, the majority were employed (84%), and most lived in an urban area (70%; Table 1). Eighteen of the 21 recognized Indigenous groups in Guatemala were represented in this sample, as well as eight of 22 possible departments (states) of residence. The average age in the sample was 29.6 years old.

**Table 1:** Sociodemographic characteristics of participants (*n* = 395).

Variables	*n* (%)
Age [mean (range)]	29.6 (18–61)
Level of education completed	
Never went to school	3 (0.8)
Elementary school	15 (3.8)
Middle school	67 (17.0)
High school	198 (50.3)
University or higher	112 (28.4)
Department/State of residence	
Alta Verapaz	23 (5.8)
Chimaltenango	17 (4.3)
Huehuetenango	27 (6.9)
Quetzaltenango	174 (44.2)
Quiché	47 (11.9)
Sacatepéquez	45 (11.4)
Sololá	36 (9.1)
Totonicapán	25 (6.3)
Indigenous group	
Achí	4 (1.0)
Akateco	2 (0.5)
Awakateco	2 (0.5)
Ch'orti'	1 (0.3)
Itzá	1 (0.3)
Ixil	3 (0.8)
Jacalteco	5 (1.3)
Kaqchikel	81 (20.6)
K'iche'	175 (44.4)
Mam	53 (13.5)
Mopan	5 (1.3)
Poqomam	1 (0.3)
Poqomchi'	5 (1.3)
Q'anjob'al	9 (2.3)
Q'eqchi'	19 (4.8)
Sakapulteco	5 (1.3)
Tz'utujil	17 (4.3)
Xinka	5 (1.3)
Employment	
Yes	328 (83.7)
No	64 (16.3)
Area of residence	
Urban	271 (69.8)
Rural	117 (30.2)

### 3.2 Results from the MIRE-Guatemala (MIRE-G) Scale

There was a high level of racism reported by participants (Table 2). 5% of participants reported high levels of interpersonal racism, and 13% reported high levels of internalized racism. More than half of participants (53.7%) reported moderate systemic racism, and another third (32.2%) reported high levels of systemic racism.

**Table 2:** Results from the MIRE-G scale.

Variable	*n* (%)
Interpersonal racism	
Composite mean (range)	1.44 (1.0–2.67)
No interpersonal racism (all responses “never”)	101 (25.5)
Low interpersonal racism (average response between “never” and “sometimes”)	273 (69.1)
16-7.4,-13.5242ptHigh interpersonal racism (average response between “sometimes” or “many times”)	21 (5.3)
Internalized racism	
Composite mean (range)	2.32 (1.25–3.0)
No internalized racism (average response “disagree”)	129 (32.7)
Low internalized racism (average response “neither agree nor disagree”)	215 (54.4)
High internalized racism (average response “agree”)	51 (12.9)
Systemic racism	
Composite mean (range)	2.08 (1.0–3.0)
Low systemic racism (average response “disagree”)	56 (14.2)
Moderate systemic racism (average response “neither agree nor disagree”)	212(53.7)
High systemic racism (average response “agree”)	127(32.2)

### 3.3 Internal validity

**Figure 1:**
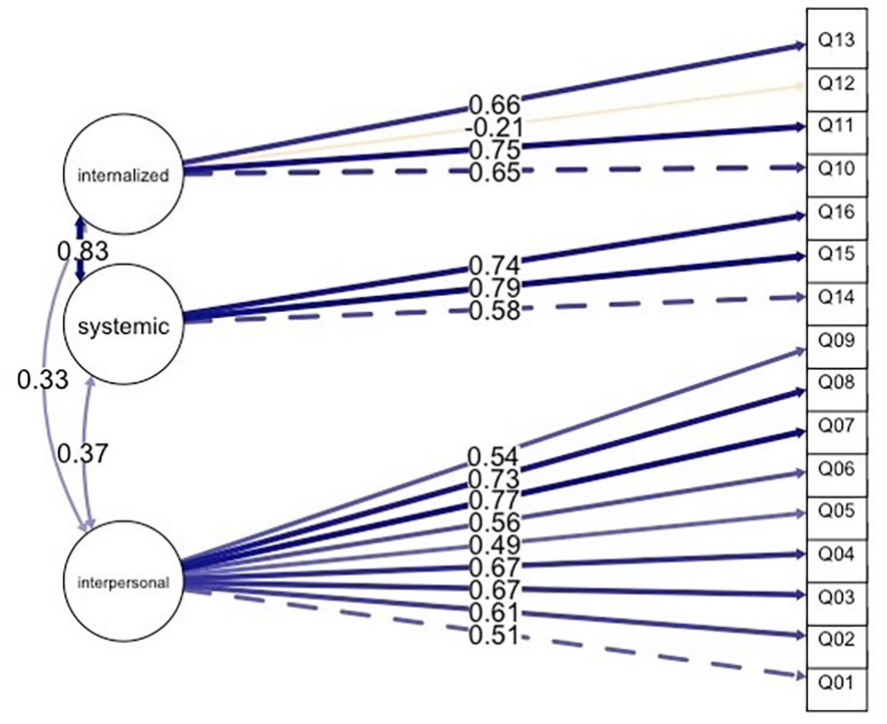
MIRE path diagram.

The original translated scale had poor fit and failed both the CFI and RMSEA tests based on the stated cut-offs (Table 3). Question Q12, assessed agreement to the statement “Indigenous people should try to think and act more like mestizos,” had a poor factor loading, indicating that the statement was not well-understood among the participants (Figure 1).

**Table 3:** Confirmatory factor analysis model fit indicators.

Indicator	MIRE	MIRE-G
Reliability		
Cronbach's alpha (Confidence Interval 95%)		
Internalized	0.522 (0.440, 0.529)	0.745 (0.660, 0.807)
Systemic	0.743 (0.686, 0.792)	0.793 (0.745, 0.832)
Interpersonal	0.847 (0.819, 0.871)	0.856 (0.829, 0.878)
CFA		
*X*^2^/df/*p*-value	382.527/101/ < 0.001	262.222/87/ < 0.001
CFI	0.847	0.901
RMSEA (Confidence Interval 90%)	0.092 (0.082, 0.101)	0.078 (0.067, 0.089)
SRMR	0.077	0.066

Due to this, the question was removed, and the CFA was re-run without the inclusion of this question. While some of the fit improved, the fit cutoffs were still not reached. As such, a modification index was performed (Table 4). The modification index indicated that Q11, “the Indigenous people have less opportunities than other Guatemalans,” originally categorized as measuring internalized racism, fit better with measurements for systemic racism. Q11 was included on the original MIRE scale to measure the factual dimension of internalized racism, whereas systemic racism questions were created to mirror common stereotypes and prejudices against Indigenous individuals (21). However, when tested on Indigenous GBM populations in Guatemala, Q11 was interpreted as a factual dimension of systemic racism by participants. Theoretically, Q11 fits in well as a measurement of systemic racism, as less opportunities for Indigenous Guatemalans could be a facet systemic racism.

**Table 4:** Modification index results.

Outcome	OP^*^	Predictor	MI^*^	EPC^*^
Systemic	= ~	Q12	21.285	−1.304
Systemic	= ~	Q11	18.257	1.747
Systemic	= ~	Q02	15.288	−0.279
Internalized	= ~	Q02	11.06	−0.248
Systemic	= ~	Q09	10.535	−0.249

^*^OP, operation; MI, Modification Index; EPC, Expected Parameter Change.

**Figure 2:**
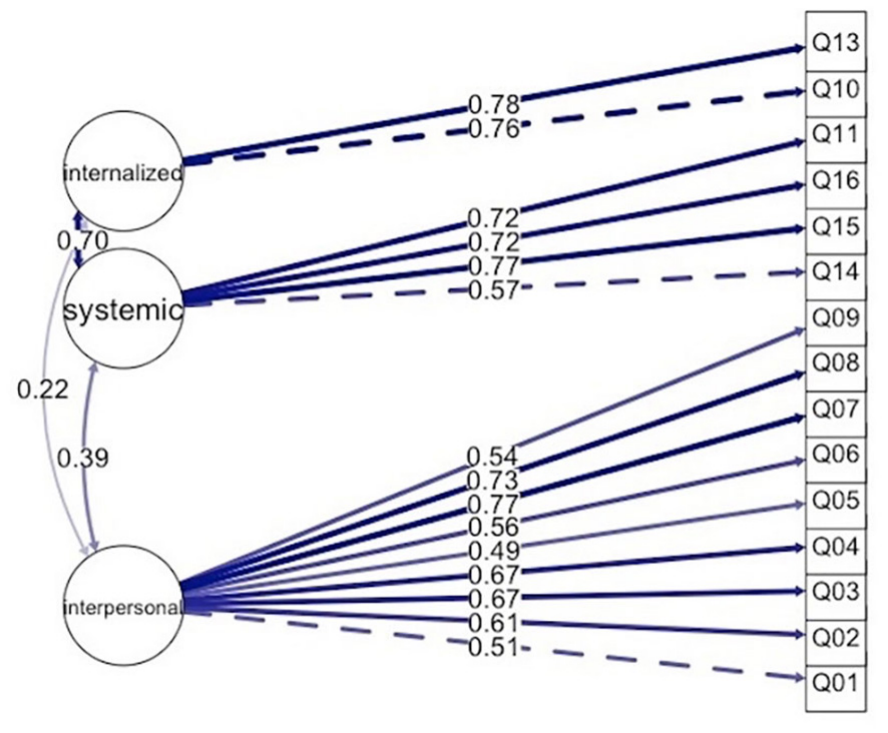
MIRE-G path diagram.

The new scale, MIRE-Guatemala (MIRE-G), which excludes Q12 and moves Q11 into the systemic racism domain, does pass all the necessary fit statistics to confirm internal validity (Table 3, Figure 2). Both the MIRE (English and Spanish versions) and the MIRE-G scales are available in Supplementary files.

### 3.4 External validity

The external validity results were mixed. Of the three hypothesized trends in the data, one was sustained with our data. Internalized racism and perception of systemic racism were inversely correlated. The hypothesis that reporting of interpersonal racism would be directly correlated with systemic racism was supported but cannot be confirmed using cross-tabulations due to cell with values less than 1. The third hypothesis, that higher reporting of interpersonal racism would be correlated with lower internalized racism failed, as the chi-square test *p*-value = 0.159.

### 3.5 Reliability

Reliability was considered adequate for the new scale as all values were above the stated cutoff at 0.70 (Table 3).

## 4 Discussion

To our knowledge, this is the first study to culturally adapt, translate to Spanish, and validate the Measure of Indigenous Racism (MIRE) for any Indigenous population in Latin America, filling an important gap. Based on the results of this study, we modified the scale for use among Indigenous GBM in Guatemala (MIRE-G). Question Q12, “Indigenous people should try to think and act more like mestizos,” was removed from the scale after factor loadings indicated that the question was poorly understood. Question Q11, “Indigenous people have less opportunities than other Guatemalans,” was moved from the internalized racism dimension to the systemic racism dimension. While the question was created to assess how Indigenous Guatemalan's felt about their own experiences, the responses from the question indicated that participants understood this question as assessing whether or not those experiences existed systemically.

Internalized and systemic racism domains were highly correlated, indicating that these dimensions of racism may not be statistically separate within this context. In the original MIRE study, Paradies and Cunningham (21) did not report the correlations between latent factors.

In terms of criterion validity of the factors, the original MIRE results followed closely with our own. Paradies and Cunningham (21) reported that internalized and systemic racism were inversely correlated within their sample, as was confirmed with our MIRE-G sample as well. Although the authors hypothesized that interpersonal racism would be associated with higher levels of systemic racism and lower levels of internalized racism, Paradies and Cunningham (21) did not find significant associations in their analysis. However, they reported that they also found no evidence that contradicted these hypotheses. In our MIRE-G study, we found supportive evidence of two hypotheses, but we could not confirm the associations between interpersonal racism and systemic/internalized racism due to limitations of the cross-tabulation analysis. The evidence of external validity therefore remains preliminary and should be expanded using other external standards in future studies.

This research has some limitations. The scale met our statistical cut-offs for construct validation; however, many of the goodness of fit tests were not strong. We used the cut-off CFI ≥ 0.90, as is considered acceptable fit, but CFI ≥ 0.95 are considered a stronger fit (28). Likewise, while RMSEA ≤ 0.08 is acceptable, RMSEA ≤ 0.06 is considered a stronger fit (28). These tests indicate that while the MIRE-G is a valid measurement of self-reported racism in Indigenous GBM, there still needs to be research and development of other scales to more accurately measure the experiences of Indigenous Guatemalans as a whole. The current study was not designed specifically for the validation of the MIRE-G scale, and as such there is an opportunity to do more focused studies on the experiences of Indigenous racism in Guatemala and Latin America. A more representative sample of Indigenous populations, ours was centered to GBM, and the inclusion of other non-MIRE variables for the purpose of improving convergent validity of the scale are both recommended. In-depth studies focused on the relationship between the three domains of racism in Latin American Indigenous GBM populations is encouraged.

This scale was developed and tested among a population of Indigenous GBM, meaning it might not be generalizable to heterosexual populations. Our team has previously shown that Indigenous GBM experience intersectional stigma due to their sexual and Indigenous identities, as well as from their gender expression, education level, and being from rural areas (10, 31). This intersectional experience may also be interacting with the independent measurement of racism. Intersectional stigma is multi-dimensional, context-dependent, and not additive across social identities, making it challenging to measure quantitatively (32, 33). Future research with Indigenous LGBTQ+ populations should consider measuring stigma based on Indigenous and LGBTQ+ identities separately and then statistically model their interactions (32).

Additionally, the survey was only translated to and administered in Spanish. While the majority of young Indigenous men (our target population) speak Spanish in Guatemala, this may have excluded individuals who only speak an Indigenous language. Future studies in Guatemala and throughout Latin America should consider translating and administering the scale in the appropriate Indigenous language.

There needs to be more research applying the new MIRE-G scale to the wider experience of Indigenous populations, expanding beyond GBM populations, both in Guatemala and in Latin America. We only validated this scale for Indigenous GBM populations in Guatemala; the MIRE-G scale is the only validated scale that measures the experiences of racism in an Indigenous population within Latin America. Indigenous groups are a large part of Latin American society, yet their specific experiences have not been adequately reflected in research. With further validation among wider Indigenous communities, the MIRE-G could be a starting point to better understand the experiences of Indigenous groups throughout Latin America, including determining the prevalence of interpersonal, systemic, and internalized racism across the region, understanding the impact of racism on mental and physical health, social wellbeing, and socio-economic status of Indigenous populations.

## 5 Conclusions

This is the first validation of the Spanish version of the MIRE scale for an Indigenous population in Latin America. The MIRE-G scale provides healthcare workers and researchers with a vigorously validated instrument to measure interpersonal, internalized, and systemic racism and its impact on health outcomes for Indigenous GBM communities in Guatemala. Additional research should be conducted to validate the MIRE-G scale with wider Guatemalan and Latin American populations, as well to understand how different dimensions of racism interact across Guatemala.

## Data Availability

The raw data supporting the conclusions of this article will be made available by the authors, without undue reservation.
